# Knowledge and Perception of Radiation Risk From Computed Tomography Scans Among Patients Attending an Emergency Department

**DOI:** 10.7759/cureus.52687

**Published:** 2024-01-21

**Authors:** Faisal H Alsubaie, Abdullah H Abujamea

**Affiliations:** 1 Department of Family and Community Medicine, King Saud University/College of Medicine, Riyadh, SAU; 2 Department of Radiology and Medical Imaging, King Saud University/College of Medicine, Riyadh, SAU

**Keywords:** perception of radiation, radiation risks, patient awareness, emergency departments, computed tomography (ct )

## Abstract

To evaluate the level of knowledge about radiation dose and possible risks related to computed tomography (CT) scans among patients visiting emergency departments (EDs), a survey was conducted over a two-month period. A total of 357 adult patients (44% men and 56% women) presenting for diagnostic imaging in the ED answered a survey consisting of 15 questions. The survey included questions about the participants' demographics and knowledge of radiation. Most of the respondents (58.5%) reported that the physician did not explain the potential risk of radiation before the procedure. In addition, more than half of the respondents (58.1%) expressed feeling anxious about the potential risk of radiation. Most respondents (84.9%) stated that the potential radiation risk did not affect their decision to proceed with the procedure. Overall, the findings highlight a lack of information about radiation and its potential risks provided to patients prior to the diagnostic procedure. Increasing awareness and understanding of the risks associated with these imaging modalities should be considered essential in modern communities. Efforts should be made to ensure that patients undergoing diagnostic imaging are aware of the radiation risks they may encounter.

## Introduction

Since its invention in the 1970s, computed tomography (CT) has significantly advanced and revolutionized diagnostic radiology, becoming crucial in diagnosing many complex diseases [1-3]. It has resulted in improved diagnosis and treatment of various diseases, faster management of trauma patients, diagnosis of pulmonary embolism (PE), improved treatment of stroke patients, better treatment of cardiac conditions, and has been extensively used in the diagnosis of patients with COVID-19 in recent years [4-7]. Compared to other imaging methods, CT offers numerous advantages; it is widely available and can be performed quickly, helping doctors confirm or rule out diagnoses with greater certainty. The utilization of CT scans in emergency departments (EDs) has dramatically increased over the past two decades, leading to increased exposure to medical radiation [8-12]. This raises concerns about radiation exposure from CT and the long-term risks of radiation-induced malignancies like radiation-induced thyroid cancer, leukemia, Hodgkin lymphoma, soft tissue sarcoma, and bone cancer. Excessive radiation exposure increases the likelihood of developing cancer [13]. Studies have shown that even at low levels, radiation exposure raises the risk of cancer [9, 14, 15]. Health organizations and authorities worldwide, such as the International Atomic Energy Agency and International Commission on Radiological Protection, have expressed concerns and emphasized the importance of controlling radiation use in medical imaging. Radiation management and guidelines have been proposed, such as the “as low as reasonably achievable” (ALARA) concept [16].

Emergency rooms see a high volume of patients for various reasons, including their availability around the clock, their proximity as the nearest point of healthcare, and their ability to provide fast accessibility without the need for distant appointments. Doctors working in emergency rooms often lack access to patients' medical and radiological histories, which forces them to consider the risk of radiation exposure against the potential of missing a life-threatening diagnosis. Several studies conducted on radiation in emergency rooms have shown that emergency care providers are concerned about radiation exposure [17-21].

In addition, studies have shown that patients underestimate the radiation from a CT scan compared to a plain X-ray and are unaware of the possible hazards of radiation-induced cancer [22-24]. However, when advanced medical imaging, particularly CT scans, is performed, patients tend to exhibit higher levels of trust in their diagnostic assessment [22].

This study aims to estimate the level of knowledge and perception about radiation risk in CT scans among adult patients attending the ED and to determine whether the level of knowledge and perceptions is affected by demographic factors.

## Materials and methods

This cross-sectional study included 357 adult patients who attended the ED at King Khalid University Hospital, Riyadh, Saudi Arabia, from December 2022 to January 2023. The study received ethical approval from the institutional review board prior to commencement (Ref. No. E-22-7379). Candidates attending the ED who underwent a CT scan and agreed to participate were provided with an electronic informational sheet explaining the purpose of the study. The questionnaire, based on one from a previous study by the author with the same objectives [25], was available in Arabic and English. It comprised 15 multiple-choice questions, including demographic information (sex, age, radiology history, and education level). Respondents were asked if they had received information about the radiation used in the procedure from the healthcare team and if this influenced their decision to continue the radiology procedure. The questionnaire also included questions about the patients' knowledge of radiation used in medical imaging and the sources of this knowledge, with options such as television and radio, newspapers and magazines, family, internet, school, friends, and the radiology technologist. Additionally, it queried which radiology modality patients believed had the highest risk, with options including CT, X-ray, or both having the same risk. The remaining questions addressed participants' knowledge of radiation, encompassing fundamental aspects of radiation safety awareness, such as the link between radiation and cancer and the cause of infertility and fetal abnormalities.

Statistical analyses were performed using IBM SPSS Statistics for Windows, Version 26 (Released 2019; IBM Corp., Armonk, New York). Quantitative variables were reported as the mean plus or minus the standard deviation, while qualitative variables were reported as proportions or percentages. Different groups were created based on gender, age group, and educational level, and responses were compared among these groups. The chi-square test was used to determine significant differences in proportions between the groups. Furthermore, a linear-by-linear association analysis was conducted to observe trends in the variables or question responses across gender, education level, and age groups. P-values less than 0.05 were considered significant.

## Results

There were 357 responses to the questionnaire, with 157 (44%) men and 200 (56%) women. The largest number of patients fell within the 21-40 age group (48.2%). Most of the respondents, 180 (50.4%), had attended university, while only 20 (5.6%) did not attend school (Table 1).

**Table 1:** Information about demographics (number, overall percentage)

Demographic information	*n *(%)
Gender	
Men	157 (44%)
Women	200 (56%)
Age range (year)	
< 21	17 (4.8%)
21–40	172 (48.2%)
41–60	124 (34.7%)
> 60	44 (12.3%)
Education	
University	180 (50.4%)
High school	104 (29.1%)
Middle school	53 (14.8%)
Did not attend school	20 (5.6%)

The majority of the respondents, 209 (58.5%), stated that the physician did not address the potential risk of radiation. Many respondents, 207 (58.1%), expressed that the potential risk of radiation made them anxious. Most respondents, 303 (84.9%), mentioned that the potential risk of radiation did not influence their decision to continue with the procedure. Additionally, 225 (63%) of the respondents reported having read or heard about the radiation risks associated with medical imaging from various sources. Only 196 (54.9%) respondents believed that CT scans have a higher risk of radiation than X-rays. More than half of the respondents, 183 (51.3%), did not believe that frequent radiation exposure could cause cancer, and only 108 (30.3%) thought that frequent radiation exposure could lead to infertility. Most of the respondents, 306 (85.7%), agreed that radiation exposure can cause fetal abnormalities (Table 2).

**Table 2:** Respondent's perception about ionizing radiation

Question	Yes, *n* (%)	No, *n* (%)
Q1. Were the potential risks of radiation explained by the doctor?	148 (41.5%)	209 (58.5)
Q2. Does the potential risks of radiation make you anxious?	207 (58.1)	150 (41.5%)
Q3. Does the potential risks affect your decision about doing the procedure?	54 (15,1%)	303 (84.9%)
Q4. Have you read or heard about the risks of medical imaging?	225 (63%)	132 (37%)
Q5. Do you think frequent exposure to radiation can cause cancer	174 (48.7%)	183 (51.3%)
Q6. Do you think frequent exposure to radiation can cause infertility	108 (30.3%)	249 (69.7%)
Q7. Do you think radiation exposure in pregnancy can cause fetal abnormalities?	306 (85.7%)	51 (14.3%)

Regarding age, we found that awareness of the radiation risk and its correlation with cancer decreases with older age (>60 years) (p-value < 0.001). Furthermore, awareness of infertility and its possibility in frequent radiation exposure were higher in the middle-aged group than in the older group (p-value = 0.004). Pregnancy risk awareness was lower in young patients (47%) compared to young adult patients (89%). Age was found to significantly impact general knowledge of radiation risk (p-value = 0.01). Young adult patients demonstrated higher levels of awareness regarding the risks associated with medical imaging compared to older patients. Moreover, we found that the potential risk of radiation was explained to the middle-aged and young-aged groups more frequently than to older people by their doctors (p-value = 0.038) (Table 3).

**Table 3:** Comparison of knowledge and awareness based on respondent's age (see Table 2 for the content of the questions).

Question	Age <20	Age 20–40	Age 41–60	Age >60	P-value
	Yes, n (%)	Yes, n (%)	Yes, n (%)	Yes, n (%)	
Q1	16 (94.1%)	58 (33.7%)	66 (53.2%)	8 (18.2%)	0.038
Q2	12 (70.6%)	82 (47.7%)	81 (65.3%)	32 (72.7%)	0.005
Q3	0 (0%)	27 (15.7%)	15 (12.1%)	12 (27.3%)	0.068
Q4	8 (47.1)	125 (72.7)	76 (61.3)	16 (36.4%)	0.01
Q5	16 (94.1)	104 (60.5%)	34 (27.4)	20 (45.5%)	<0.001
Q6	4 (23.5%)	64 (37.2%)	36 (29%)	4 (9.1%)	0.004
Q7	8 (47.1%)	153 (89%)	109 (87.9%)	36 (81.8)	0.249

Regarding education level, we found that doctors explained the potential risk of radiation more often to patients with high school degrees than those with bachelor's degrees (p-value = 0.009). Furthermore, we found that general knowledge about the possible risk associated with radiation increased with the level of education (p-value = <0.001). However, we observed no significant relationship between awareness of radiation risk of causing cancer and education level. In addition, education level significantly impacted awareness of the correlation between frequent radiation exposure and infertility (p-value = 0.001). Additionally, education level was significantly associated with awareness of pregnancy risks (p-value = 0.002) (Table 4).

**Table 4:** Comparison of knowledge and awareness based on education level (see Table 2 for the content of the questions).

Question	University	High school	Middle school	Did not attend school	P-value
	Yes, n (%)	Yes, n (%)	Yes, n (%)	Yes, n (%)	
Q1	50 (27.8%)	66 (63.5%)	28 (52.8%)	4 (20%)	0.009
Q2	98 (54.4%)	61 (58.7%)	36 (67.9%)	12 (60%)	0.14
Q3	23 (12.8%)	27 (26%)	4 (7.5%)	0 (0%)	0.341
Q4	132 (73.3 %)	69 (66.3%)	16 (30.2%)	8 (40 %)	<0.001
Q5	84 (46.7%)	62 (59.6%)	20 (37.7%)	8 (40%)	0.513
Q6	68 (37.8%)	36 (34.6%)	4 (7.5%)	0 (0%)	<0.001
Q7	165 (91.7%)	85 (81.7%)	40 (75.5%)	16 (80%)	0.002

**Figure 1:**
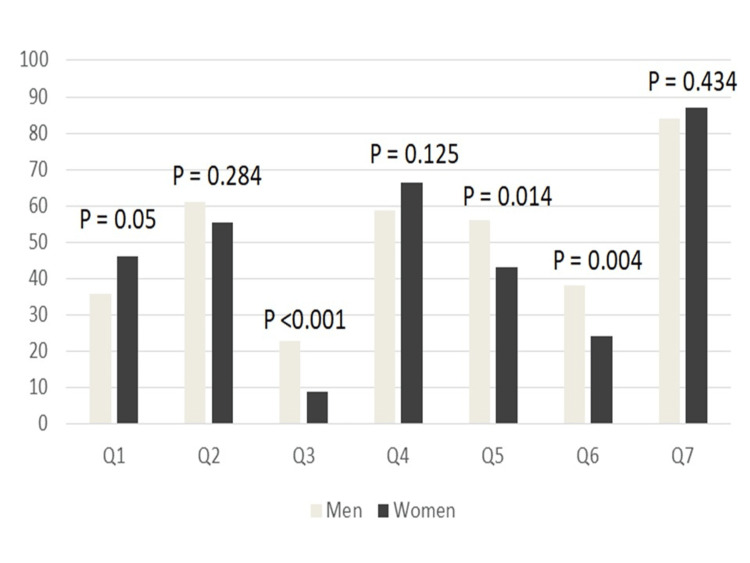
Perception of radiation risk based on gender Comparison of gender-wise patient awareness of ionizing radiation (see Table 2 for the content of the questions).

Regarding gender, we found that the potential risk significantly impacted men's decisions about undergoing the procedure (p-value < 0.001). However, general knowledge about the possible risks was not significantly related to gender. Men were more likely than women to believe that radiation exposure correlated with cancer (p-value = 0.014). Furthermore, men had a significantly higher belief than women that radiation exposure could increase the risk of infertility (p-value = 0.004). Gender had no impact on awareness of pregnancy risks (Figure 1).

## Discussion

In this study, we found that only 48.7% of respondents believed that frequent radiation exposure could increase cancer risk. Moreover, only 54.9% of the participants thought that CT scans were more dangerous than X-rays. These findings highlight the importance of adequately informing patients about the benefits and risks of radiation in medical imaging. Our study variables included age, gender, and educational level. While our study had a limited sample size, it accurately reflects our population in terms of age and gender. It also includes different levels of education and can provide a comprehensive awareness assessment among patients visiting EDs. Our results go in parallel with the findings of the Takakuwa study, which highlighted the role of educational level in radiation-associated risk awareness; patients with a higher level of education had more knowledge about radiation-associated risks [24]. Regarding the possibility of radiation-induced infertility and fetal abnormalities, awareness was higher in the group with higher education levels. Similar to our previous study, we found that the perception of potential radiation-induced infertility was undoubtedly influenced by gender [25]. Unfortunately, women were less informed of radiation risks and their impact on fertility. In addition, unlike our previous study, we found a difference in awareness of radiation risk and its correlation with cancer between men and women, with men exhibiting higher awareness than women [25]. Regarding the adverse effects of radiation on pregnancy, 85.7% of our respondents believed that radiation exposure during pregnancy increases the risk of fetal abnormalities, exceeding the 49.3% found in the Aldossari study [26].

Our survey results showed that young and elderly adult age groups, as opposed to middle-aged groups, had a relatively low degree of knowledge and awareness of radiation risks. Therefore, more attention should be given to health education to raise awareness among these age groups. Although the study's findings indicate that more than half of the participants (58.5%) in the survey were not adequately informed about the radiation dosage and potential dangers associated with their CT scan, this is still lower than what was reported by Lee et al., who found that 93% of patients did not receive such information from their doctors and the emergency team [27]. This indicates a lack of communication between physicians and patients. In modern medical practice, an important aspect is shared decision-making, which involves patients in the decision-making process regarding the need for medical imaging and possible adverse effects. In the emergency room, providing all the details about a diagnostic test can be challenging because of the patient's condition. Patients understand that discussions with physicians are constrained by time limitations. In EDs, doctors might think that it is not necessary to discuss the small risk of imaging against the significant risk of an incorrect diagnosis. Furthermore, some doctors may avoid discussing radiation risks because they think that the patients might decline the study [28]. An effective framework for discussing CT radiation risk is informed decision-making, which gives patients the option to accept a diagnosis and opens up a dialogue about radiation exposure while considering the patient-physician relationship [29,30]. Keijzers and Britton found that emergency physicians are more likely to discuss radiation risks with patients who are at higher risk of radiation exposure, such as pediatrics and pregnant patients [31]. Lam et al. found that part of the miscommunication of radiation risk to patients is due to physicians having limited knowledge about radiation risk from CT scans and the estimation of the average effective dose from CT investigations compared to chest radiography [32].

Moreover, significant institutional variation exists regarding who should inform patients about radiation doses and potential risks. While referring physicians were more likely to inform patients about why they needed imaging, technicians and radiologists were more likely to explain the procedure and any possible risks to patients at the time of imaging [33,34]. While this study offers valuable insights within our hospital context, its generalizability is limited by the small sample size and focus on one institution. Future research with larger, diverse samples from multiple hospitals could strengthen our understanding and provide more broadly applicable results.

## Conclusions

The role of diagnostic imaging in the healthcare system has been quickly evolving in recent years. Although it significantly improves clinical practice and patient management, it may be accompanied by potential health risks. This study aimed to assess the awareness of radiation risks from CT among our study population. Despite the limited sample size of this study, we found a significant number of patients lack a comprehensive understanding of radiation in medical imaging. Awareness and understanding of the risks involved with these imaging modalities should be essential components of modern communities. Enhancing patient understanding of risks and benefits through open communication, along with continuous education of healthcare professionals on alternative diagnostic options, can empower informed choices and minimize unnecessary radiation exposure.
